# *In Vivo* Vascularization of Endothelial Cells Derived from Bone Marrow Mesenchymal Stem Cells in SCID Mouse Model

**DOI:** 10.22074/cellj.2016.4312

**Published:** 2016-05-30

**Authors:** Abdolamir Allameh, Maryam Jazayeri, Maryam Adelipour

**Affiliations:** 1 Department of Clinical Biochemistry, Faculty of Medical Sciences, Tarbiat Modares University, Tehran, Iran; 2 Department of Biochemistry, School of Medical Sciences, Iran University of Medical Sciences, Tehran, Iran

**Keywords:** Mesenchymal Stem Cell, Endothelial Cells, Cell Transplantation, Differentiation

## Abstract

**Objective:**

*In vivo* and *in vitro* stem cell differentiation into endothelial cells is a promising area of research for tissue engineering and cell therapy.

**Materials and Methods:**

We induced human mesenchymal stem cells (MSCs) to differentiate to endothelial cells that had the ability to form capillaries on an extracellular matrix (ECM) gel. Thereafter, the differentiated endothelial cells at early stage were characterized by expression of specific markers such as von Willebrand factor (vWF), vascular endothelial growth factor (VEGF) receptor 2, and CD31. In this experimental model, the endothelial cells were transplanted into the groins of severe combined immunodeficiency (SCID) mice. After 30 days, we obtained tissue biopsies from the transplantation sites. Biopsies were processed for histopathological and double immunohistochemistry (DIHC) staining.

**Results:**

Endothelial cells at the early stage of differentiation expressed endothelial markers. Hematoxylin and eosin (H&E) staining, in addition to DIHC demonstrated homing of the endothelial cells that underwent vascularization in the injected site.

**Conclusion:**

The data clearly showed that endothelial cells at the early stage of differentiation underwent neovascularization *in vivo* in SCID mice. Endothelial cells at their early stage of differentiation have been proven to be efficient for treatment of diseases with impaired vasculogenesis.

## Introduction

Endothelial cells can be differentiated from bone marrow derived hematopoietic stem cells (HSCs), endothelial progenitor cells (EPCs), mononuclear cells (MNCs) and mesenchymal stem cells (MSCs) (1). MSCs possess the ability to differentiate into endothelial cells in the presence of an appropriate stimulating factor such as vascular endothelial growth factor (VEGF) and insulin-like growth factor (IGF-1) (2,6). IGF-1 upregulates the expression of CXC chemokine receptor 4 (CXCR4) as a migratory factor (7,8). Developing endothelial cells are often associated with expression of endothelial markers such as von Willebrand factor (vWF), VEGF receptors 1 (VEGFR1/FLT-1) and 2 (VEGFR2/KDR), Tie2, CD31,VE-cadherin, and vascular cell adhesion protein 1 (VCAM-1) (9,12). Differentiation of bone marrow-derived MSCs into endothelial cells and the ability of the cells for *in vitro* capillary network formation have been examined on a semi-solid gel matrix (4,8). 

EPCs that have the capacity for angiogenesis and vasculogenesis were successfully used for therapeutic angiogenesis (stimulation of angiogenesis) of ischemic diseases. In this case, the increasing vascularity and improving cardiac function in ischemic myocardium and reconstitution of the blood brain barrier (BBB) in stroke has been reported (13,15). Tsukada et al. (16) reported the effects of two types of EPC (small-EPC and largeEPC) in a hindlimb ischemia model on *in vivo* neovascularization. They showed that the largeEPC promoted neovascularization in the murine hindlimb ischemia model. 

Human EPCs were used to improve blood flow recovery and capillary density in ischemic hindlimbs of nude mice (17). Kawamoto et al. (18) transplanted human EPCs into Hsd:RH-rnu (athymic nude) rat models of myocardial ischemia and reported markedly improved capillary density. They used immunohistochemistry analysis to show the presence of capillaries that were positive for human-specific endothelial cells. 

The therapeutic potential of EPC for cell therapy of injured blood vessels and prosthetic grafts was reported by Griese et al. (19). EPC transplanted into balloon-injured carotid arteries and bioprosthetic grafts in rabbits resulted in rapid endothelialization of the denuded vessels and graft segments. A study reported the induction of angiogenesis and myogenesis in an acute myocardial infarction rat model following administration of MSCs (20). According to Wei et al. (21), MSCs placed in hypoxic conditions prior to their transplantation caused enhancement of angiogenesis in a cerebral ischemia rat model. 

We reported the earlier differentiation potential of human MSCs into capillaries on a matrigel (8). The developing vascular cells that recovered under this condition possessed molecular and cellular characteristics of endothelial cells. In the present study, we sought to determine whether MSCs at the early stage of differentiation to endothelial cells could efficiently form a vessel network in a mouse model. The differentiated cells were injected into the groins of severe combined immunodeficiency (SCID) mice in order to evaluate their efficiency to induce *in vivo* angiogenesis. 

## Materials and Methods

### Isolation of human bone marrow mesenchymal stem cells

Bone marrow aspiration was collected from five healthy donors (age 20-49 years) at the Bone Marrow Transplantation Center, Shariati Hospital, Tehran, Iran. Each patient provided informed consent prior to collection of the samples. The experimental part of the study was carried out in accordance with a protocol approved by Tarbiat Modares University Medical Ethics Committee. 

MSCs were isolated using Ficoll-Hypac (Biochrom, Germany). The bone marrow sample (7-10 ml) was layered on top of a Ficoll-Hypac (d=1.077 g/ml) and centrifuged at 2200 rpm for 20 minutes at room temperature. The interface layer that contained MNCs was collected and washed twice in phosphate-buffered saline (PBS, Gibco, USA). Next, in order to culture the cells, we placed them in 25 cm^2^ flasks that contained Dulbecco’s modified eagle’s medium-high glucose (DMEM-HG, Gibco, USA) supplemented with 10% fetal bovine serum (FBS, Gibco Invitrogen, USA), 2 mM GlutaMAX-I™ (L-alanyl-L-glutamine, Gibco Invitrogen, USA), 10 U/ml penicillin and 100 mg/ml streptomycin (Biochrom, Germany). Cells were incubated at 37˚C in 5% CO_2_ . The non-adherent cells were removed after 24 hours by washing the seeded cells with PBS and changing the medium. The medium was changed every 3 days until the cells reached 80-90% confluence. The MSCs were recovered using 0.25% trypsin-EDTA (Biochrom, Germany) and replated at 5000-6000 cells per cm^2^ of the flask’s surface area and considered as passage 1 (P1) cells. 

### Differentiation of the mesenchymal stem cells to osteocytes and adipocytes

We verified the differentiation potential of MSCs to osteocytes and adipocytes. Differentiation to adipocytes was induced by culturing MSCs in DMEM supplemented with 10% FBS, 1 mM dexamethasone (Sigma, USA), 200 mM indomethacin (Sigma, USA), 1.7 mM insulin (Sigma, USA), 500 mM isobutyl-methylxanthine (Merck, Germany), 0.05 U/ml penicillin, and 0.05 mg/ml streptomycin for 2 weeks. We have used oil red-O staining (Sigma, USA) to identify the presence of adipocytes. Oil red-O staining visualizes intracellular lipid accumulation. For staining, the cells were fixed in 10% formaldehyde (Merck, Germany) for 1 hour, after which they were washed with 60% isopropanol, (Merck, Germany) and stained with oil red-O solution in 60% isopropanol for 10 minutes. Next, cells were washed with distilled water and de-stained in 100% isopropanol for 15 minutes. 

Differentiation of MSCs to osteocytes was induced Stem Cell Derived Endothelial Cells for Neovascularization in α-MEM (Gibco, USA) that contained 10% FBS, 0.1 mM dexamethasone, 10 mM β-glycerophosphate (Sigma, USA), and 50 mM ascorbate-phosphate (Sigma, USA) for two weeks. A specific histochemical stain for alkaline phosphatase (ALP) with an alkaline phosphatase staining kit (Sigma Chemical Co., USA) was used to identify the osteocytes. 

### In vitro induction of mesenchymal stem cell differentiation to endothelial cells

We cultured confluent passage 3 MSCs in medium complete with trace elements 131 (MCDB131, Sigma Chemical Co., USA) that contained 5% FBS, 50 ng/ ml VEGF and 20 ng/ml IGF-1 (Peprotech, USA), and incubated them at 37˚C for 5 days. During this period the medium was changed twice per week. 

### Immunophenotyping of mesenchymal stem cells and endothelial cells

We used trypsin (0.05%) and EDTA (0.02%) to remove the MSCs and differentiated endothelial cells from the culture flasks. The cells were counted with a Neubauer slide and cell viability determined with trypan blue staining. The cell suspension (10^6^ cells/ml) was prepared in 50 μl PBS and incubated with either fluorescein isothiocyanate (FITC) or PE-conjugated antibodies, in the dark for 45 minutes at 4˚C. Immud nophenotyping of the MSCs was carried out using anti-CD44-PE, anti-CD166-PE, anti-CD105-FITC, and anti-CD34-FITC antibodies (eBioscience, USA). In order to immunophenotype the endothelial cells before and after differentiation, we incubated the MSCs and differentiated endothelial cells with anti-FLT1FITC, anti-VE-cadherin-FITC, anti-VCAM1-FITC, anti-Tie2-FITC, anti-vWF-FITC, anti-CD31-FITC, and anti-VEGFR2-FITC antibodies (eBioscience, USA). Next, the cells were washed twice with PBS that contained 2% bovine serum albumin (BSA, Gibco Invitrogen, USA) and fixed with 1% paraformaldehyde solution in PBS. Mouse isotype antibodies served as the negative control. Analysis was performed using a flow cytometer (Partech, Germany). 

### Reverse transcriptase-polymerase chain reaction

We assessed the expressions of the endothelial specific gene markers *CD31, VWF, VEGFR2* and *FLT1* by reverse transcriptase-polymerase chain reaction (RT-PCR). Total RNA was extracted from the differentiated endothelial cells using guanidine thiocyanate (Merck, Germany). The samples were subjected to a reverse transcriptase (RT) reaction to synthesize the first cDNA strand using a cDNA synthesis kit (Takara, USA). The cDNA synthesis was performed with 500 ng total RNA, 20 pmol oligo dT primer, 0.5 mM dNTP mixture, 5X PrimeScript Buffer, 20 units RNase inhibitor, 100 units PrimeScript reverse transcriptase, and RNase-free dH_2_O to 20 μl according to the manufacturer’s protocol. RT-PCR was performed using PCR buffer (Qiagen, USA) in a 50 μl reaction mixture that contained 1 μL of first-strand cDNA, 0.5 U of recombinant Taq DNA polymerase, 1.5 mM MgCl_2_, 0.2 mmol/L dNTPs, and 40 pmol of the following primers for:

VEGFR2
  F: 5ʹ-TGGCATGGTCTTCTGTGAAG-3ʹ
  R: 5ʹ-AATACCAGTGGATGTGATGCG-3ʹ
VWF
  F: 5ʹ-AATGTTGTGGGAGATGTTTGC-3ʹ
  R: 5ʹ-GTGGATATCCACCTCTACTTCAGAC-3ʹ
FLT1
  F: 5ʹ-CGACCTTGGTTGTGGCTGACT-3ʹ
  R: 5ʹ-ACCCTTCTGGTTGGTGGCTTTG-3ʹ
CD31
  F: 5ʹ-AACAACGAGAAAATGTCAGATCC-3ʹ
  R: 5ʹ-GGAGCCTTCCGTTCTAGAGT-3
and GAPDH as the housekeeping gene
  F: 5ʹ-CTCTCTGCTCCTCCTGTTCG-3ʹ
  R: 5ʹ-ACGACCAAATCCGTTGACTC-3ʹ

The PCR profile consisted of 5 minutes of initial denaturation at 94˚C, followed by 25 cycles of ded naturation for 30 seconds at 94˚C, 60 seconds of annealing at 53-60˚C, 45 seconds of extension at 72˚C, and a final extension step of 10 minutes. An aliquot of the PCR product (20 μl) was separated by electrophoresis on a 2% agarose gel and stained with ethidium bromide. 

### In vivo vascularization assay

This experimental study was carried out on five SCID mice (6-8 weeks old). The animals were purchased from Pasteur Institute of Iran and maintained under sterile conditions. In order to identify transplanted cells in the vessel form, the endothelial cells at early stage of differentiation (day 5) were labeled using a 1:1000 dilution of bromodeoxyuridine (BrdU, Sigma, USA). After 24 hours, the cells were washed with PBS and suspended in MCDB-131 medium (100 μl) that contained 2% FBS. Then, the SCID mice received subcutaneous injections of 10^6^ differentiated endothelial cells Allameh et al. into their left groins. Simultaneously, the same volume of MCDB-131 medium that contained no cells was injected into the right groins of the animals, as the control. 

At 30 days after the cell transplantation, we obtained tissue biopsies from the injection sites (left and right groins) and the peritoneum of each mouse. Tissues were processed for hematoxylin and eosin (H&E) and double immunohistochemistry (DIHC) staining. 

### Double immunohistochemistry

Histological sections were prepared from paraffin-embedded biopsy samples collected from each cell transplantation site. The histological sections were located on polylysine-coated plus glass slides. After deparaffinization with xylene (Merck, Germany) and ethanol (Merck, Germany), the tissue sections were autoclaved at 100˚C for 20 minutes in a citrate buffer (pH=6) for antigen retrieval. After the addition of normal goat serum as a protein blocker on the blank sites of the tissue sections, the tissues were subjected to double labeling. Briefly, the primary antibody against the BrdU marker (anti-BrdU antibody, cat. ab8955, Abcam) diluted with PBS (2:100) was added to the tissue sections for one hour. After washing with PBS, the biotin-HRP-labeled anti-mouse IgG (secondary antibody) was applied for 30 minutes and the reaction was detected with diaminobenzidine (DAB) as a chromogen, followed by the addition of potassium ferricyanide (Merck, Germany), a stabilizer for horseradish peroxidase. The sections were washed with PBS and incubated for one hour with primary antibody against the vWF marker (anti-vWF antibody, ab49706, Abcam) diluted with PBS (5:100). After washing the slides with PBS, the biotin-HRP-labeled anti-mouse IgG (secondary antibody) was applied for 30 minutes, and amino ethyl-carbazol (AEC) was added as a chromogen. Then, oxidation of DAB and AEC were performed using horseradish peroxidase in the presence of H_2_O_2_ Finally, hematoxylin staining was used as the background staining. For each preparation, we performed H&E and DIHC staining of BrdU and vWF in order to trace the transplanted endothelial cells. 

## Results

### Characterization of human bone marrow mesenchymal stem cells

**Fig.1:**
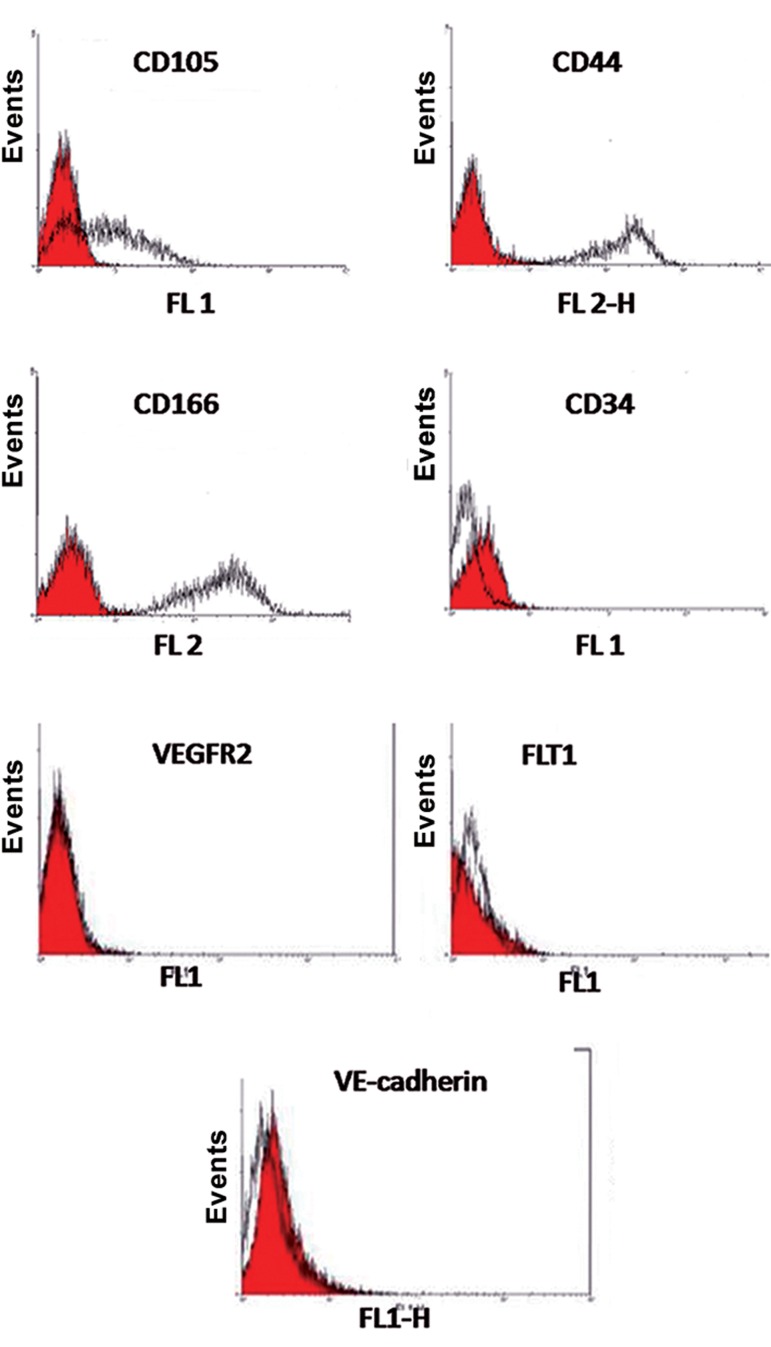
Characterization of mesenchymal stem cells (MSCs) by flow cytometry. MSCs were negative for CD34 (hematopoietic marker) and positive for CD44, CD105 and CD166. The shaded area shows the profile for the negative control and MSCs were negative for the endothelial markers vascular endothelial growth receptor 2 (VEGFR2), FLT1 and VE-cadherin.

Flow cytometric assays showed that the MSCs prior to differentiation expressed CD105, CD44, and CD166 as surface markers. There was no expression of CD34 as an early hematopoietic marker in these cells. MSCs did not express the endothelial cell markers VEGFR2, FLT1, and VEcadherin (Fig.1). 

### Differentiation potential of mesenchymal stem cells into adipocytes and osteoblasts 

**Fig.2:**
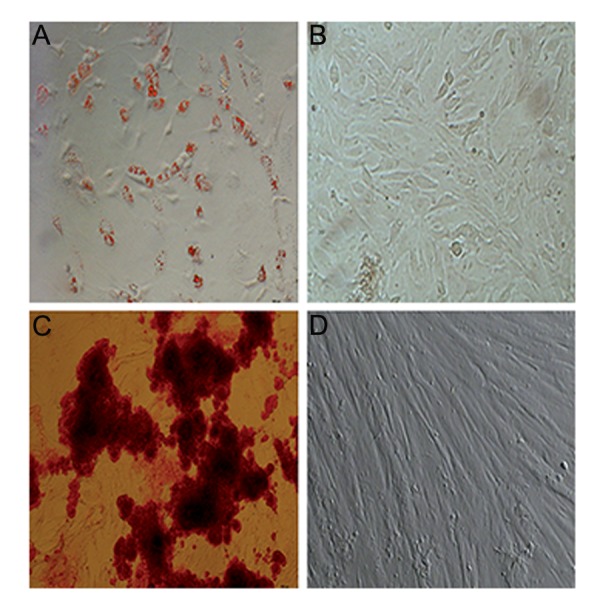
Characterization of mesenchymal stem cells (MSCs) by their ability to differentiate into adipocytes and osteocytes. A. The results of oil red-O staining in adipocytes that differentiated from MSCs, B. Negative control for adipocytes, C. The results of alkaline phosphatase (ALP) staining in osteocytes that differentiated from MSCs, and D. Negative control for osteocytes (magnification: ×10).

We confirmed the differentiation potential of MSCs by inducing their differentiation into adipocytes in a specific differentiation medium. Adipocytes derived from MSCs on day 14 of differentiation had a round shape that contained various fat vacuoles in their cytoplasm. More than 80% of all cells stained by oil red-O-stain 14 days after induction of differentiation (Fig.2A). However there were no fat droplets observed in the undifferentiated MSCs (Fig.2B). 

Likewise, we assessed the ability of these MSCs to differentiate into osteoblasts by specific histochemical staining of the differentiated osteoblasts for ALP. 

The majority of MSCs (90%) were ALP positive (Fig.2C). Untreated MSCs were negative for spontaneous osteoblast formation even after 3 weeks of cultivation (Fig.2D). 

### Characterization of the differentiated endothelial cells

**Fig.3:**
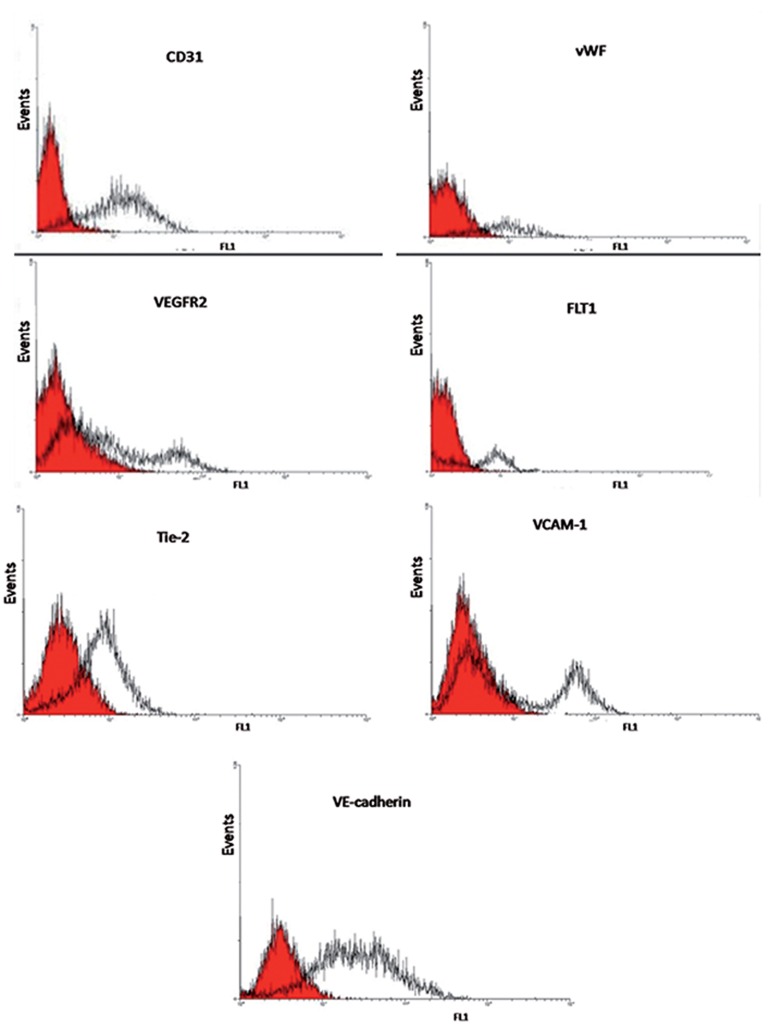
Flow cytometry characterization of the endothelial cells derived from mesenchymal stem cells (MSCs) for endothelial markers. These cells were positive for CD31, von Willebrand factor (vWF), vascular endothelial growth factor receptor 2 (VEGFR2), FLT-1, Tie2, vascular cell adhesion protein 1 (VCAM-1), and VE-cadherin.

RT-PCR and flow cytometric assays confirmed the presence of differentiated endothelial cells. Flow cytometry data showed that the endothelial cells derived from MSCs expressed *CD31, vWF, VEGFR2, FLT1, Tie2, VCAM1* and *VE-cadherin* as specific endothelial markers (Fig.3). 

**Fig.4:**
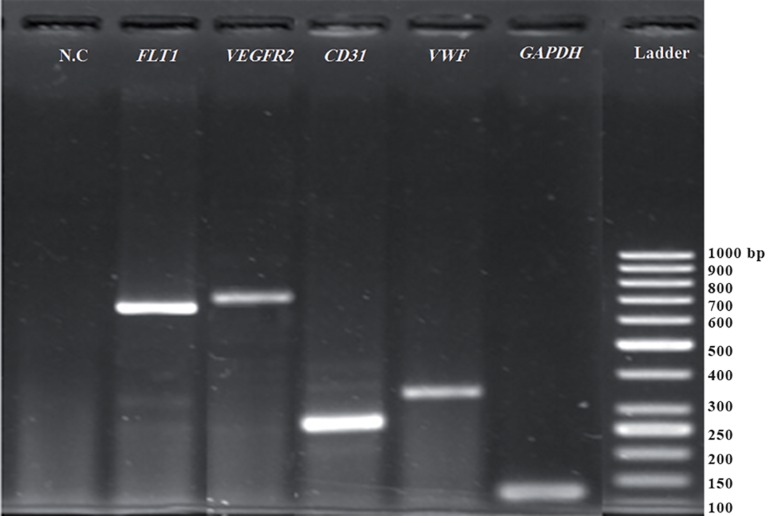
Expression of selected endothelial specific genes by RT-PCR. Endothelial cells derived from mesenchymal stem cells (MSCs) expressed vascular endothelial growth factor receptor 2 (VEGFR2), CD31, Von Willebrand factor (VWF) and FLT-1. *GAPDH* was considered to be the housekeeping gene. N.C; Negative control.

RT-PCR data showed that the differentiated endothelial cells expressed the endothelial specific genes *CD31, VWF, VEGFR2,* and *FLT1* (Fig.4). 

The endothelial cells derived from MSCs expressed VEGFR2 as shown by flow cytometry from two days after differentiation until day five of differentiation. 

### In vivo vascularization of the differentiated endothelial cells 

**Fig.5:**
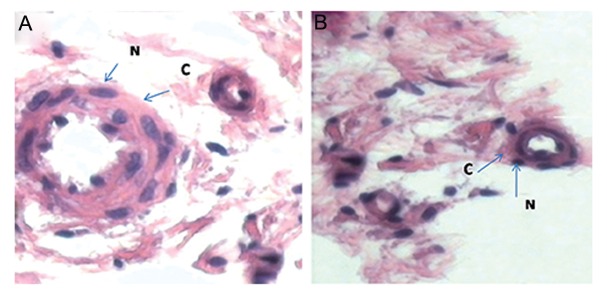
Formation and development of neovascular structures in cell-treated groins of mice compared with the control groins (H&E). A. Tissue from the cell treatment site and B. Tissue from the control site. C; Cytoplasm of the endothelial cells that participated in angiogenesis stained a red color and N; Nucleus of the endothelial cells that participated in angiogenesis and stained violet (magnification: ×80).

**Fig.6:**
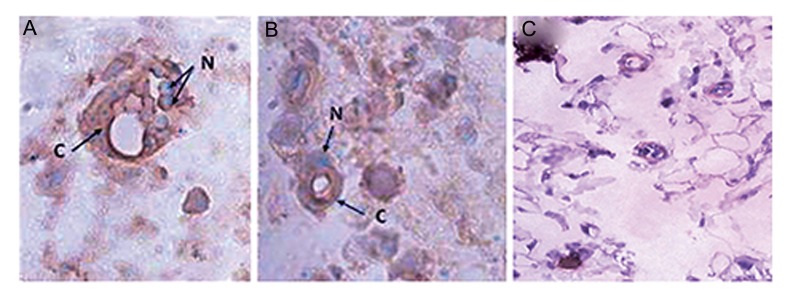
Contribution of endothelial cells derived from mesenchymal stem cells (MSCs) in the formation of a neovascular structure with double immunohistochemistry (DIHC) staining. The heterologous differentiated endothelial cells incorporate into sites of neovasculariza- tion. A, B. Tissue from the cell treatment site and C. Tissue from the control site. c; Cytoplasm of the endothelial cells that participated in angiogenesis stained red and N; Nuclei of BrdU-labeled endothelial cells that participated in angiogenesis stained light green (magnifica- tion: ×200).

The endothelial cells derived from MSCs underwent angiogenesis in five SCID mice, as shown by H&E and DIHC staining. H&E staining of a histological section taken from the endothelial cell injection sites (left groin) showed vessel formation compared to the samples taken from the right groin, as the control that received an injection of culture medium and no cells (Fig.5). DIHC results showed the participation of differentiated endothelial cells in angiogenesis. Treated samples contained endothelial cells with green nuclei and red cytoplasm, which were considered positive for BrdU (green nuclei) and vWF (red cytoplasm, Fig.6). This finding indicated that the differentiated endothelial cells pretreated with BrdU participated in angiogenesis in the SCID mice. The endothelial cells in the normal tissues were positive only for vWF (cytoplasm is stained in red and nuclei are stained in violet). 

## Discussion

Impaired angiogenic balance can lead to angiogenesis-dependent disorders. Hyperangiogenesis is often observed in some disorders such as tumor progression. Hypoangiogenesis occurs in cases where there is insufficient vascularization such as ischemic heart disease (22,26). Therefore, angiogenesis is considered a therapeutic target for a variety of diseases. Proor anti-angiogenesis agents are a topic of angiogenesis research (23,24). In recent years, progenitor stem cells have been isolated and directed towards formation of endothelial cells which have the capability to form capillaries. Most studies show that the endothelial cells isolated from stem cells can develop into capillaries on synthetic and semi-synthetic matrix enriched with VEGF and other stimulating factors (8). However, the efficiency and the condition for the endothelial cells to identify the disrupted endothelial network and to contribute to their angiogenesis are not well understood. 

The advantage of the present study was the use of endothelial cells at the early stage of differentiation in order to observe their contribution to the formation of a new vessel network in a mouse model. 

Various groups of researchers reported the development of capillary density in animal impaired angiogenesis models after EPC transplantation. According to Kalka et al. (17), hEPCs were used for enhancing blood flow recovery and capillary density in ischemic hindlimb mice. Kawamoto et al. (18) reported the transplantation of hEPCs to an Hsd:RH-rnu (athymic nude) rat model of myocardial ischemia. They observed significant improvement in capillary density. Rapid endothelialization of denuded vessels and graft segments in a rabbit model of balloon-injured carotid arteries and bioprosthetic grafts after EPC therapy was reported by Griese et al. (19). A large EPC was shown to promote neovascularization in a murine hindlimb ischemia model as reported by Tsukada et al. (16). Nagaya et al. (20) observed enrichment of angiogenesis and myogenesis in a rat model of myocardial infarction following MSCs therapy. Enhancement of angiogenesis using hypoxic preconditioning of MSCs therapy in a cerebral ischemia rat model was reported by Wei et al. (21). 

Earlier, we discussed the ability of MSCs isolated from human bone marrow to induce endothelial cells and promote capillary network formation on an extracellular matrix (ECM) (8). The *in vitro* development of capillaries on the synthetic ECM was clearly shown. The developmental changes in the endothelial cells into capillaries was demonstrated by transmission electron microscopy (TEM) (9). 

The application of endothelial cells in the early stage of differentiation for cell transplantation and involvement in angiogenesis was unclear for us. A number of studies have shown *in vivo* angiogenesis induction by transplantation of progenitor stem cells. The major issues are the selection of the stem cell type and the protocol used to prepare the cells prior to transplantation. Considering these parameters, in the present study human MSCs have been isolated and differentiated into endothelial cells. The availability, fast growth and ability to differentiate into endothelial cells by MSCs were additional advantages of the current experiment. We chose cells at their early stage of differentiation (day 5) for *in vivo* transplantation into the SCID mice model to avoid immune response reactions. The groin region of the animals was chosen as the site of cell transplantation and angiogenesis development. Under the study protocol, the cells on day 5 of differentiation possessed the typical morphological and molecular characteristics of endothelial cells. Immunophenotyping of the cells showed expression of endothelial markers VEGFR-2, VE-cadherin, VCam-1 and vWF together with morphological changes (microvessel formation in ECM) in the cells. These factors indicated the endothelial induction of MSCs. 

The histopathological observations of biopsies of the stem cell transplanted region revealed the development of new capillaries. According to the results of nuclear staining by BrdU, incorporation of the stain showed the formation of new endothelial cells. The formation of neovascular structures in the injection site was verified by histopathological studies as well as the double staining technique performed on tissue biopsies. The 30 day period after cell transplantation was considered in order to visualize the changes in the sprouts of the microvascular structures in the region. The site of cell transplantation (groin of the SCID mice) chosen in the present study was advantageous, particularly due to the accessibility of the tissue for treatment and sampling. The optimization stage of the endothelial differentiation and transplantation might be implicated in treatment of diseases associated with angiogenesis complications. 

The limited data provided in this article together with our previous results suggest that MSCs derived endothelial cells under optimized conditions can contribute to *in vivo* neovascularization. 

## Conclusion

The endothelial cells at their early stage of differentiation from MSCs show neo-vascularization and angiogenesis potential in SCID mouse. 
